# A new immunotherapy strategy targeted CD30 in peripheral T-cell lymphomas: CAR-modified T-cell therapy based on CD30 mAb

**DOI:** 10.1038/s41417-021-00295-8

**Published:** 2021-01-29

**Authors:** Yang Wu, Dan Chen, Ya Lu, Shu-Chen Dong, Rong Ma, Wei-yan Tang, Jian-qiu Wu, Ji-Feng Feng, Jian-Zhong Wu

**Affiliations:** 1 Research Center of Clinical Oncology, Jiangsu Cancer Hospital & Jiangsu Institute of Cancer Research & Nanjing Medical University Affiliated Cancer Hospital, Nanjing, 210009 P. R. China; 2 Research Center of Clinical Oncology, Nanjing Medical University Affiliated Cancer Hospital, Nanjing, 210009 P. R. China; 3 Department of Medical Oncology, Jiangsu Cancer Hospital & Jiangsu Institute of Cancer Research & Nanjing Medical University Affiliated Cancer Hospital, Nanjing, 210009 P. R. China

**Keywords:** Cancer therapy, Cancer

## Abstract

Chimeric antigen receptor T-cell immunotherapy (CAR-T) has shown remarkable efficacy in treating tumors of lymphopoietic origin. Herein, we demonstrate an effective CAR-T cell treatment for recurrent and malignant CD30-positive peripheral T-cell lymphomas (PTCL) has been demonstrated. The extracellular fragment gene sequences of CD30 were obtained from tumor tissues of PTCL patients and cloned into a plasmid vector to express the CD30 antigen. The CD30 targeting single-chain antibody fragment (scFv) was obtained from CD30-positive monoclonal hybridoma cells, which were obtained from CD30 antigen immunized mice. After a second-generation of CAR lentiviral construction, CD30 CAR T cells were produced and used to determine the cytotoxicity of this construct toward Karpas 299 cells. The results of CD30 CAR T-mediated cell lysis show that 9C11-2 CAR T cells could significantly promote the lysis of CD30-positive Karpas 299 cells in both LDH and real-time cell electronic sensing (RTCA) assays. In vivo data show that 9C11-2 CAR T cells effectively suppress the tumor growth in a Karpas 299 cell xenograft NCG mouse model. The CD30 CAR T cells exhibited an efficient cytotoxic effect after being co-cultured with the target cells and they also exhibited a significant tumor-inhibiting ability after being intravenously injected into PTCL xenograft tumors; these observations suggest that the new CD30 CAR-T cell may be a promising therapeutic candidate for cancer therapy.

## Introduction

Peripheral T-cell lymphoma (PTCL) is a type of malignant lymphoma derived from T cells at different stages of thymus development, with biological behavior and clinical manifestations of heterogeneity [1, 2]. As the incidence of PTCL has increased year to year, the diagnosis and treatment of PTCL are receiving more and more attention. PTCL accounts for approximately 23–27% of non-Hodgkin’s lymphoma (NHL) in China [3, 4], which is much higher than the 10–15% observed in Western countries [5], and shows significant heterogeneity [6]. PTCL represents a relatively rare heterogeneous NHL group with a very poor prognosis [7, 8]. Conventional chemotherapy based on anthracyclines is still the standard treatment, but most patients have difficulty achieving satisfactory results after initial treatment or during final relapse treatment [9], and the 5-year survival rate of patients with PTCL is only 38.5% [10, 11]. Therefore, the discovery of new and effective treatment strategies for PTCL is of the utmost importance for clinicians and patients of this form of NHL.

In-depth research of tumor biology and immunology has increased significantly in recent years, and immunotherapy has gradually become one of the leading trends for cancer treatment, providing new ideas and new methods for the effective treatment of lymphomas [12–15]. CAR-T cells targeting CD19 or CD20 have achieved remarkable results in the treatment of acute hepatitis B leukemia, chronic lymphocytic leukemia, and NHL [16–20], indicating that CAR-T has attractive clinical application prospects for lymphoma treatment. Many studies have found that CD30 is highly expressed in 50% of PTCL patients [21–24]. Until recently, two drugs have been used to target CD30, and they have had positive effects on patients with CD30-positive Hodgkin’s lymphoma (HL) [25–27]. Although CD30 is an effective target for lymphoma therapy, reports of CAR-T cell therapy targeting PTCL based on CD30 mAb remain limited [28].

In this study, we describe the development of a novel CD30 hybridoma obtained from a CD30 antigen immunized mouse. Furthermore, we constructed three kinds of CD30 lentiviral CARs to induce CD30 CAR T. Then, we chose the most effective lentiviral system to significantly suppress the growth of CD30-positive PTCL cells (Karpas 299) in vitro and in vivo. The use of mature CAR-T cell immunotherapy outlined in this study undoubtedly provides a new CAR-T cell therapy based on anti-CD 30 mAb for the treatment of refractory or recurrent PTCL.

## Material and method

### Cell lines

The human anaplastic large cell lymphoma Karpas 299 cell lines were purchased from the American Type Culture Collection (ATCC, USA). Karpas-299 were cultured in Roswell Park Memorial Institute medium (RPMI-1640, Gibco, USA) supplemented with 10% fetal bovine serum (FBS, Wisent Biotechnology, Canada), and maintained in a 5% CO_2_ incubator with a 37 °C humidified atmosphere. Karpas 299 cell line was authenticated by STR and tested mycoplasma contamination before experiments.

### Expression and purification of CD30 recombinant proteins

A total of 20 tissue samples were collected from patients with CD30-positive PTCL recruited from Nanjing Medical University Affiliated Tumor Hospital. The study was approved by the institutional research ethics committee of Nanjing Medical University Affiliated Tumor Hospital, and written informed consent was obtained from each subject. The nucleotide sequences encoding the extracellular domain of CD30 from CD30-positive patients (tested by IHC, ab203593 Abcam) were obtained by reverse transcription-polymerase chain reaction (PCR) amplification. After detection by electrophoresis using a 1% agarose gel, the fragment was inserted into the BamHI and HindIII restriction sites of the prokaryotic expression vector pET-28a. The recombinant plasmid was then transformed into *Escherichia coli* DH5α competent cells with calcium chloride. After overnight incubation, the positive colonies were selected with kanamycin (100 μg/mL, Sigma, USA). Finally, the plasmid DNA from positive colonies was extracted using a Plasmid Mini Kit (QIAGEN, Germany) and identified by restriction enzyme digestion and sequence confirmation PCR with the following primers: (forward primer 5′-CGCGGATCCGGCACGGCGCAGAAGAACAC-3′, reverse primer 5′-CCCAAGCTTTTACTTCCCCGTGGAGGAGAG-3′). Each sample was tested three times.

Recombinant CD30 proteins were expressed by transforming the recombinant plasmid pET28a-CD30 into *E. coli* DH5α cells incubated in LB medium with kanamycin (100 μg/mL, Sigma, USA). After induction for 4 h with 3 µM isopropyl-β-d-thiogalactoside (IPTG, Thermofisher, USA) at 37 °C, the target proteins were purified by Ni-NTA agarose from the culture medium. The purified CD30 protein was verified using Western blot analysis and mass spectrum identification.

### Preparation and selection of CD30 hybridoma

Five Balb/c mice (male, 7 weeks old, GemPharmatech, China) were immunized with multipoint-subcutaneous injections of 500 μL CD30 antigen (50 μg) emulsified in Freund’s complete adjuvant. Immunizations were conducted every 2 weeks for a total of three immunizations. Mouse serum was collected on the fourth day after the third immunization for indirect enzyme-linked immunosorbent assay (ELISA) analysis, and 50 μg of CD30 antigen protein in Freund’s incomplete adjuvant was injected into the caudal vein and the intraperitoneal to strengthen the immunity of the mice. In order to generate hybridoma cells, spleen cells collected from immunized mice were fused with SP/0 myeloma cells at a ratio of 5:1 using polyethylene glycol solution (PEG_2000_, Sigma, USA) and then cultured in hypoxanthine (HAT) medium (DMEM supplemented with 20% FBS and 2% HAT, Gibco, USA) with 5% CO_2_. After 2 weeks, monoclonal hybridomas were diffused in 96-well plates and the positive clones were selected by indirect ELISA to further propagate for the production of CD30 monoclonal antibodies. The accumulation and purification of monoclonal antibodies from culture supernatants were obtained from a protein G column (Thermofisher, USA). Each sample was tested 3 times. The specificity of CD30 antibody from CD30 hybridoma was assayed by flow cytometry, immunofluorescence, western blot, and immunohistochemical.

### Synthesis of scFv obtained from CD30 hybridoma cells

The gene sequences of the CD30 scFv antibody were obtained from 9C11 monoclonal hybridoma cells as discussed below in Section “Results”. We used the protocol of the Mouse scFv Extraction and Assembly Kit (PPL, China) for scFv extraction. Briefly, 1 × 10^7^ monoclonal hybridoma cells were collected by centrifugation and total RNA was extracted using the RNeasy kit (Qiagen, USA). Single-stranded cDNA was synthesized from total RNA using the RT master mix (Takara, China). Two pairs of primers were used to amplify the VH and VL genes from cDNA. PCR products were separated on a 1% agarose gel and the bands at 350 bp were excised and purified using a gel extraction kit. Purified VH and VK, as well as (G4S)_3_, were used as templates in overlapping PCR. After PCR amplification, VH and VL were linked by (G4S)_3_ to form the ScFv expression cassette. Each sample was tested three times.

### CD30 CAR lentiviral construction and lentivirus production

The scFv of CD30 mAb, which specifically recognizes the CD30 antigen, was synthesized and sub-cloned in-frame into the lentiviral backbone. The genetic sequence of the second-generation CD30 CAR was comprised of CD8aSP (a signal peptide from the CD8 family), CD30 scFv, human CD8 hinge following the specific scFv as a transmembrane domain, co-stimulator 4-1BB, and the CD3ζ signaling domain used to activate CAR-T cells. The positive clone was further confirmed by DNA sequencing and termed pCDH-CD30.CAR-GFP. Each sample was tested three times.

Lentiviral particles were generated by co-transfection of CD30 CAR lentivirus plasmids (pCDH-CD30-CAR-GFP) with packaging plasmids pMD2 and psPAX2 (Addgene, USA) into HEK-293T cells using Lipofectamine 2000 (Life Technologies, USA) according to the manufacturer’s instructions. Forty-eight hours post transfection, supernatants were collected, filtered, and concentrated using PEG it^™^ Virus Precipitation Solution (Stratech Scientific Ltd., UK). Finally, the products were stored at −80 °C and the virus titer was measured as 3 × 10^8^ (TU/mL). Each sample was tested three times.

### PBMC collection and CD30 CAR-T cells induced

All specimens were collected under a protocol approved by the Nanjing Medical university-affiliated Cancer Hospital review board, and written informed consent was obtained from each donor. Peripheral blood mononuclear cells (PBMCs) were isolated from venous blood samples within 4 h using lymphocyte separation medium (MP biomedicals, France) and were centrifuged using a standard density gradient separation method at 400 g for 20 min at room temperature. PBMCs were then washed and resuspended in GT-T551 H3 medium (TaKaRa, China) containing 100 ng/mL CD3 (TaKaRa, China), 100 ng/mL CD28 (Novoprotein, China), and 500 U/mL IL-2 (Novoprotein, China). After cell counting, PBMCs were seeded in a 6-well flat-bottom plate at a concentration of 5 × 10^6^ cells/mL and cultured in a cell incubator at 37 °C with a humidified atmosphere and 5% CO_2_.

To obtain the CAR-T cells, 1 × 10^7^ PBMCs were collected and infected by CD30 CAR lentivirus (CD30 CAR T) or non-effective CAR lentivirus (NC CAR T) at a multiplicity of infection of 5 and 8 μg/mL polybrene (Sigma, USA), respectively. During the 14 days culture, the proliferation of CAR-T cells was monitored using a cell counter (Countess II FL, Life, USA) and a fluorescence microscope (Vert.A1, Zeiss, USA) every week. The CD3-, CD4-, CD8-, and CD30-positive cell ratios were evaluated by flow cytometer (Canto, BD, USA). Samples were mixed with staining reagents including anti-human CD3 PE (Biolegend, 300308), anti-human CD4 PerCP-Cy5.5 (Biolegend, 317428), and anti-human CD8 APC (Biolegend, 344722). After 30 min incubation in the dark, cells were washed and resuspended in phosphate-buffered saline (PBS) solution and analyzed using a multi-color flow cytometer (FACS Canto, BD USA). For analysis, a first gate using SSC/CD3-PE characteristics of CD3-positive lymphocytes was defined. Then, from these cells, CD4, CD8, and CD30 expression were gated and analyzed PerCP-Cy5.5/APC dot plot or on a simple histogram (count/fluorescein isothiocyanate). Each sample was tested three times.

### In vitro cytotoxicity of CAR-T cells redirected to CD30 on peripheral T-cell lymphomas cells

To test the cytotoxicity of CAR T cells, and lactate dehydrogenase (LDH) assay was used to assess the level of apoptosis in the target cells. The LDH release assay was carried out using the LDH cytotoxicity assay kit (Promega, USA) to determine the killing ability of three kinds of CD30 CAR T cells in vitro. Typically, two CD30-positive PTCL cell lines (Karpas 299 and SU-DHL-1, 1 × 10^4^ cells) and CD30-negative cell lines (Jurkat, 1 × 10^4^ cells) were respectively cocultured with effector cells of CD30 CAR-T cells or NC CAR T cells in GT551 medium at effector cells: target cells (E:T) ratios of 40:1, 20:1, and 10:1. After incubation for 24 h, 50 μL of cellular supernatant was obtained from each sample by centrifugation (400 g, 5 min). The cellular supernatant was transferred to a new 96-well plate and 50 μL of the reaction mixture (assay buffer stock solution and the substrate stock solution) was incubated in each well at room temperature for 30 min. Following this, 50 µL of stop solution was added to the mixture, and the 96-well plates were placed into a microplate reader (Biorad, USA) to obtain the absorbance value at 490 nm. Karpas 299 cells were treated with 10 μL of cell lysis buffer for 45 min to detect their maximum-release LDH activity. Each sample was tested three times.

A cytotoxicity assay was performed to determine whether 9C11-2 (abbreviated as CD30) CAR T cells could specifically recognize and kill CD30-positive PTCL cells. Briefly, the target Karpas 299 cells were seeded in the xCelligence E-plates of a real-time cell electronic sensing assay station (RTCA, ACEA biosciences, USA) at an abundance of 1 × 10^5^ cells per well. The 9C11-2 CAR T cells were then added into each well at E:T ratios of 40:1, 20:1, and 10:1. Cell proliferation was monitored by measuring the conductivity of the cell surface area in contact with the gold electrodes covering the plate surface for 24 h after cell addition. Each sample was tested three times.

The apoptosis rate of the target cells was also measured by flow cytometry. The Karpas 299 cells were collected and washed with fresh RPMI 1640 medium, then 2 μL of cell membrane labeling dye (PKH26, sigma, USA) was added to the 1 × 10^7^/mL Karpas 299 cells. After 5 min of incubation, 1 mL FBS was added to the Karpas 299 cells to stop the staining, and the cells were washed twice with a fresh GT551 medium. The labeled Karpas 299 cell were cocultured with the effector CAR T cells at different E:T ratios for 24 h and the cells were collected and 2 *μ*L cell viability staining solution (7-AAD, eBioscience, USA) was added to identify dead cells. After 5 min of incubation at room temperature, cells were washed twice with PBS and immediately analyzed by flow cytometer. The target cells were set to include all PKH-26 labeled cells in FL2, and the FL4 fluorescence from these cells was displayed as a histogram. The data collection was terminated after 1 × 10^4^ PKH-26 labeled cells were acquired by flow cytometry. The cytotoxicity of CAR T cells was expressed as a percentage of cell death within the PKH26-positive target cells. Each sample was tested three times.

The IL-2, IFN-γ, and TNF-α secreted by the 9C11-2 CAR T cells were measured using the MILLIPLEX MAP Human CD8^+^ T Cell Magnetic Bead Panel (Millipore, USA) on the FLEXMAP 3D system (Luminex, USA). Typically, 1 × 10^4^ Karpas 299 cells were suspended in 100 μL GT551 and seeded in a 96-well plate, and the CD30 CAR T cells were added at different E:T ratios. After a 24 h coculture, 100 μL of cellular supernatant was collected for measurement of cytokines. Moreover, whether soluble CD30 antigen can blocks the CD30 CAR T cells attack toward CD30-positive tumor cells was further investigated. Each sample was tested three times.

### In vivo activity of CAR-T cells redirected to CD30 on peripheral T-cell lymphomas

To investigate the CD30 CAR T cell responses against CD30-positive human PTCL tumor in vivo, we subcutaneously injected 2 × 10^6^ Karpas 299 cells (suspended in 100 μL PBS with 100 μL matrigel (Life sciences, USA)) into the right flank of NOD-Prkdc^em26Cd52^Il2rg^em26Cd22^/Nju mice (female, 7 weeks old, NCG, GemPharmatech, China) to produce a tumor xenograft model. Twenty-four mice were random divided into three groups when tumor sizes reached 50 mm^3^, then mice were then intravenously injected with 1 × 10^7^ CD30 CAR T cells, 1 × 10^7^ NC CAR T cells, or saline for each group. The tumor dimensions were monitored by calipers and the tumor volumes were calculated as the length of tumor × width^2^ of tumor/2. After 30 days, mice were sacrificed for analysis of tumor tissue. The TNF-α, INF-γ, and GM-CSF levels in tumor tissue were measured by immunohistochemistry (IHC) analyses. The survival rates of the mice and CAR T cells in peripheral blood were also assessed. Peripheral blood was obtained by retro-orbital bleeding and the CD3- or CD30-positive cell percentage rate was quantified using a flow cytometer (Canto, BD, USA). All in vivo mouse experiments were approved by the Nanjing Medical University animal care committee. Each sample was tested three times.

### Statistical analysis

Statistical analysis was performed using the SPSS 19.0 software for Windows (SPSS Inc., Chicago, IL). Chi-square test was used for comparison of mice survival rates between the CD30 CART group and the Saline group. Differences were considered to be statistically significant at *p* value less than 0.05, and the *p* value less than 0.01 was considered as very significant.

## Results

### Expression and purification of CD30 recombinant proteins

**Fig. 1:**
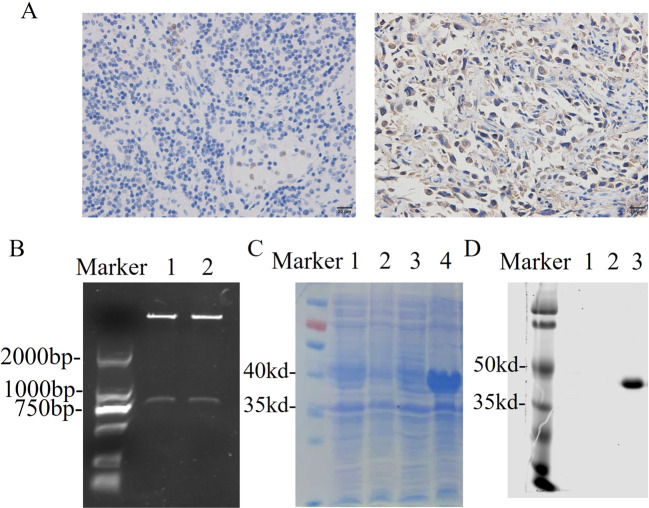
Construction and expression of the recombinant CD30 antigen protein. **A** Immunohistochemical assay of a CD30-positive biopsy sample (left CD30^−^, right CD30^+^); **B** gel electrophoresis assay of the recombinant pET28a-CD30 construct (1, 2) plasmid, **C** the coomassie blue staining of the CD30 antigen; (1. Negative control without IPTG, 2. Negative control with IPTG, 3. pET28a-CD30 without IPTG, 4. pET28a-CD30 with IPTG), **D** Western blot assay of CD30 antigen (1. Negative control with IPTG, 2. pET28a-CD30 without IPTG, 3. pET28a-CD30 with IPTG).

The expression of the CD30 antigen in PTCL tumor tissue was shown by IHC, and the results revealed that CD30-positive PTCL tissue had much more brown or light brown staining (Fig. 1A, right) than CD30-negative tissue (Fig. 1A, left). The extracellular segment of the CD30 protein was obtained by cloning the CD30 sequence into the pET328a plasmid vector. Gel electrophoresis results showed that the BamHI and HindIII digested pET328a-CD30 plasmid produced a band at 1000 bp (Fig. 1B), and the CD30 band was verified by sequencing (Fig. S1). The SDS-PAGE results showed that the CD30 antigen was successfully expressed in *E. coli* BL21 cells after induction with IPTG (Fig. 1C, D).

### Preparation and selection of anti-CD30 hybridoma

**Fig. 2:**
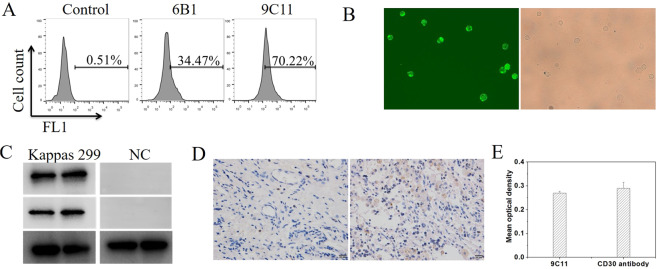
Characterization of CD30-specific monoclonal antibody. **A** Flow cytometry assay the specificity of CD30 antibody from 6B1 and 9C11 monoclonal hybridoma cells, **B** immunofluoresence assay the specificity of CD30 antibody at 20× amplification, **C** Western blot assay the specificity of CD30 antibody (9C11, middle line), Abcam CD 30 antibody (up line) and GAPDH (bottom line) used as a control, **D**, **E** immunohistochemical assay the specificity of CD30 antibody.

The titer of the mouse antiserum and the positive monoclonal antibody of hybridomas, which secreted mAb against the CD30 antigen, were detected by ELISA. The cultured supernatants of 9C11 hybridomas had much higher OD values than other cultured hybridoma supernatants (Table S1). The results of immunofluorescence assay (IFA) and flow cytometry demonstrated that the CD30 antibody purified from the cultured supernatant of 9C11 hybridomas could specifically combine with the CD30-positive Karpas 299 cells. (Fig. 2A, B). Furthermore, the western blotting result (Fig. 2C) showed that the CD30 antibody could specifically combine the CD30 antigen in the same manner as the Human anti-CD30 antibody (ab203593, Abcam). Moreover, the IHC test also confirmed that the purified CD30 antibody from 9C11 hybridomas could specifically combine with CD30-positive tissue (Fig. 2D, right). Figure 2E shown that the mean optical density of CD30-positive tissue shown no significant difference between purified CD30 antibody and human anti-CD30 antibody (ab203593, Abcam). These results confirm purified CD30 antibodies from 9C11 hybridomas can specifically recognize CD30-positive PTCL tissue.

### scFv obtained from CD30 hybridoma cells and synthesized

**Fig. 3:**
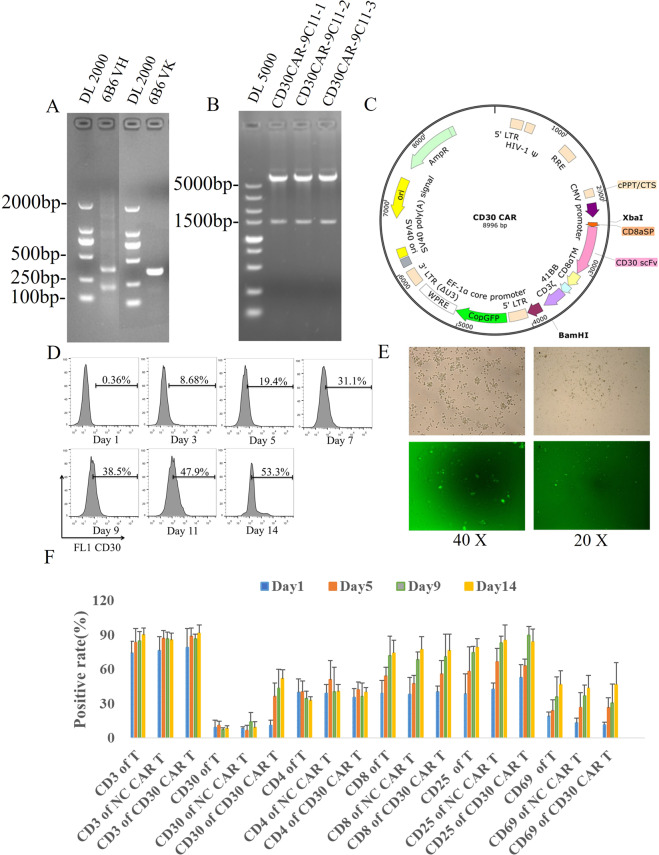
Synthesized of CD30 CAR and prepared CD30 CAR T cell. **A** Gel electrophoresis assay of VH and VL genes from monoclonal hybridoma cells, **B** restriction enzyme analysis of the CD30-specific recombinant plasmid, **C** the diagram of CD30-specific recombinant plasmid. **D** Generation and proliferation of CD30 CAR T cells (9C11-2 CAR-T) and flow cytometer assay of the CD30 expression rate of 9C11-2 CAR T cells over 14 days, **E** fluorescence microscopy monitoring of CD30 CAR T cells, **F**, **G** CD3, CD4, CD8, and CD30 expression rates of CD30 CAR T cells over 14 days.

To obtain the sequences of the CD30 scFv, two fragments coding VH or VL were amplified from the 9C11 hybridoma cell line. The linker between VH and VL was (G4S)3, and the leading sequence was derived from human CD8a. As shown in Fig. 3A, the CD30 scFv possesses three complementary determining regions (CDRs). These sequences are typically present in the amino acid sequences of murine IgG. Identification of three CDRs (9C11-1, 9C11-2, and 9C11-3) in each variable region indicate that CD30 scFv encoded by the amplified gene products could be classified into the murine antibody repertoire (Table S2 and 3).

### CD30 CAR lentiviral construction and lentivirus production

The genetic sequence of the second-generation CD30 CAR was comprised of CD8Asp (a signal peptide from the CD8 family), CD30 scFv, human CD8 hinge (following the specific scFv as the transmembrane domain), one co-stimulator 4-1BB to transduce intracellular signaling, and a CD3ζ signaling domain to activate CAR-T cells (Fig. 3B, C). The construction of the CD30 CAR expression vector was verified by an XbaI and BamHI double enzyme digestion assay. The 1% agarose gel electrophoresis result showed that the size of three CD30 CAR sequences was about 1000 bp and the sequencing results confirmed CD30 CAR sequences were consistent with expectations.

### PBMC collection and CD30 CAR-T cells induced

The generation and proliferation of CD30-specific CAR T cells were measured by flow cytometry, fluorescence microscopy, and a cell counter, during in 14 days culture. Figure S2 shows that the number of CD30 CAR T cells or NC CAR T cells increased with the number of days, and the number of CD30 CAR T cells and NC CAR T cells increased 9.7 ± 2.64 and 10.3 ± 2.68 fold after being cultured for 14 days. Furthermore, the CAR T-cell activation rate and the CD30 expression rate were demonstrated through CD3 (Biolegend, USA), CD4 (Biolegend, USA), CD8 (Biolegend, USA), and F(ab’) (Sigma, USA) fragment expression in CAR T cells. As the results show, lentiviral gene-modified CD30 CAR T cells successfully expressed CD30 on their surface, and the expression increased from 11.2% on day 1 to 51.7% on day 14 (Fig. 3F). The CD8^+^, CD25^+^, and CD69^+^ CAR T-cell populations had a higher degree of proliferation in vitro as the culture progressed. The percentage of the CD8^+^ cell subset expanded from 40.3% on day 1 to 76.2% on day 14, and the percentage of the CD25^+^ cell subset expanded from 52.7 to 83.8% on day 14 (Fig. 3F). Moreover, the generation, proliferation, and apoptosis of T cell, NC CART cell, or CD30 CART cell were monitored by flow cytometry (Figs. S2 and 3).

### In vitro cytotoxicity of CAR-T cells redirected to CD30 on peripheral T-cell lymphoma cells

**Fig. 4:**
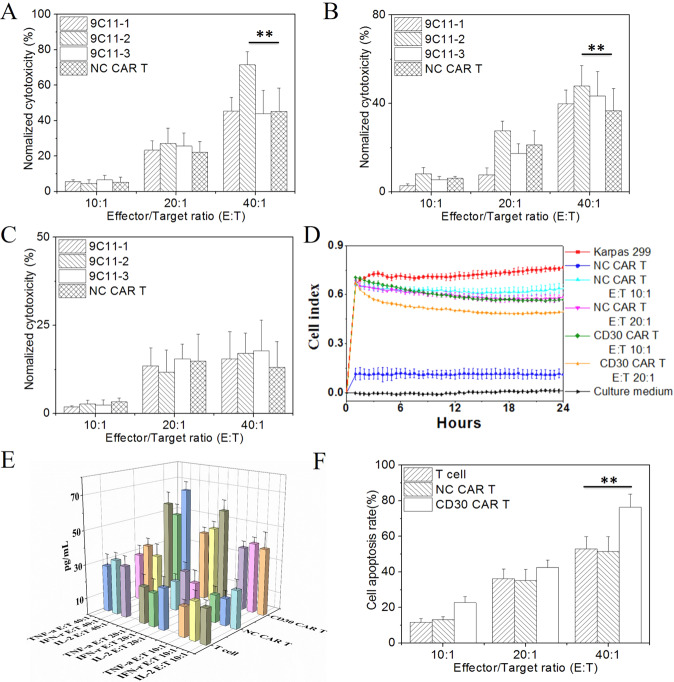
In vitro cytotoxicity of CD30 CAR-T cells. The LDH assay of cytotoxicity of CD30 CAR T cells to Karpas 299 (**A** CD30^+^, **p < 0.01 vs. NC CART), SU-DHL-1 (**B** CD30^+^, **p < 0^.^01 vs. NC CART) and Jurkat (**C** CD30^−^); **D** the RTCA assay of the impedance of Karpas 299 cells, the reduction in impedance of Karpas 299 cells means more cells were killed by CD30 CAR T cells at E:T of 20:1, **E** the cytokine release of CD30 CAR T cells measured by Luminex 3D, **F** flow cytometry was used to evaluate the apoptosis rate of Karpas 299 cells, **p < 0.01 vs. NC CART.

To test how the three kinds of CAR T-specific CD30s mediated cell lysis, an LDH assay was used to measure the degree of injury to target cells (CD30-positive PTCL cell lines, Karpas 299 and SU-DHL-1; CD30-negative cell lines, Jurkat). The results show that the cytotoxicity of effector cells increased with an increasing E:T ratio (Fig. 4A–C), and CD30 CAR T cells could effectively kill CD30-positive PTCL cell lines. The cytotoxicity of the 9C11-2 group showed significant differences compared to the other three groups when the E:T ratio was 20:1 and 40:1 in the karpas 299 cell line. Furthermore, the cytotoxicity rate of the 9C11-2 CAR T cells increased to 86.5% when the E:T ratio was 40:1. This result may contribute to the ability of 9C11-2 CAR T cells to effectively recognize the CD30 antigen of Karpas 299 cells, and we further used Karpas 299 cells as the target cell line.

Furthermore, the cytotoxic of target 9C11-2 CAR T cells was monitored by RTCA; the impedance of each well was recorded to compare the cytotoxic CAR T-cell activity required to distinguish between different E:T ratios of CAR T cells. In this assay, target CD30 CAR T cells were cocultured with Karpas 299 at E:T ratios of 10:1,20:1 and 40:1; the NC CAR T cells were used as a negative control. As shown in Fig. 4B, the impedance of each well was monitored over 24 h and the results demonstrate that T cells produced modest impedance, while the Karpas 299 cells showed strong and increased impedance, which is consistent with rapidly proliferating cells. In the 9C11-2 CAR T cell and Karpas 299 cell coculture group, the reduced impedance was dependent on the increased E:T ratio. Figure 4B shows that there is a significant difference between the lytic activity of the target CD30 CAR T cell group as compared to the NC CAR T cell group (*p* < 0.01). The T cells stimulated by anti CD3 and anti CD28 showed low-level lytic activity. The CAR T cells could be able to kill target tumor cells through either an antigen-specific or non-specific Fas:Fas ligand interaction function to release granzymes and perforins. The results show that these two approaches work synergistically to kill target tumor cells and the method appears to be useful for multiple targeted tumor cells.

Moreover, 9C11-2 CAR T cells were activated upon engagement of CD30-positive cancer cells, as indicated by the release of cytokines. In the cytokine release assay, IL-2, IFN-γ, and TNF-α were measured by FLEXMAP 3D at E:T ratios of 40:1, 20:1, and 10:1. Figure 4C shows that IL-2, IFN-γ, and TNF-α were produced in greater amounts by target CD30 CAR-T cells than NC CAR-T cells or T cells when cocultured with CD30-positive Karpas 299 cells at E:T 40:1 (*p* < 0.05). Cytokine levels in the cell supernatant increased when CD30 CAR T cells were incubated with Karpas 299 cells in a CAR T cell dose-dependent manner. There were no significant differences in IL-2, IFN-γ, and TNF-α levels between NC CAR T cells and T cells after incubation with target cells at E:T ratios of 40:1, 20:1, and 10:1. This result indicates that the increase in cytokine release is dependent on CD30 CAR signaling. We further investigated whether soluble CD30 affects the CD30 CAR T-cell attack toward kappas 299 cells. The result shows that IL-2 levels at different E:T ratios (Fig. S2, F) have no significate difference in present or in absence of soluble CD30 (20 μg/mL, Jingbai Biotech, China). This result indicated that the soluble CD30 did not block activation of CD30 CAR T cells toward kappas 299 cells.

To investigate cell-mediated cytotoxicity, the cell membrane fluorochrome PKH-26 was used to label Karpas 299 cells, and the DNA intercalating dye 7-AAD was used to label nonviable Karpas 299 cells. The results show that the cell apoptosis rate increased with an increasing E:T ratio (Fig. 4D). Notably, CD30 CAR T cells possess significantly better killing ability than NC CAR T or T cells.

### In vivo activity of CAR-T cells redirected to CD30 on peripheral T cell lymphomas

**Fig. 5:**
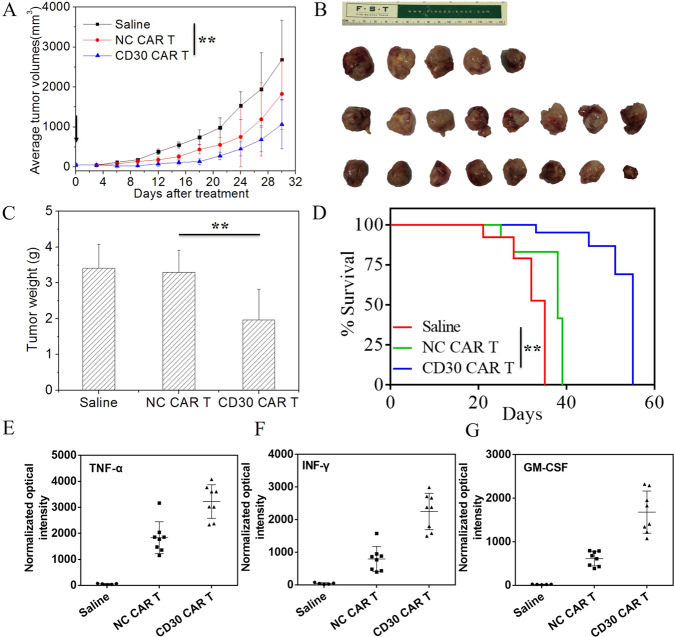
In vivo xenograft tumor suppression by CD30 CAR T cells. **A** The average tumor dimensions were monitor after intravenously injected with CD30 CAR T cells, NC CAR T cells, or saline at the day of 1 (arrow show, **p < 0.01 vs. saline), **B** the imaging of tumor tissue treated with CD30 CAR T (bottom line), NC CAR T (middle line), and saline (up to line) at 30 days, **C** the tumor weight at 30 days, **p < 0.01 vs. saline, **D** the survival rate of xenograft tumor-bearing mice, **p < 0.01 vs. saline, immunohistochemical analysis of cytokine levels in tumor sections at day 30, GM-CSF (**E**), TNF-α (**F**), and INF-γ (**G**) expression in tumors is shown as percentages of positive area.

**Fig. 6:**
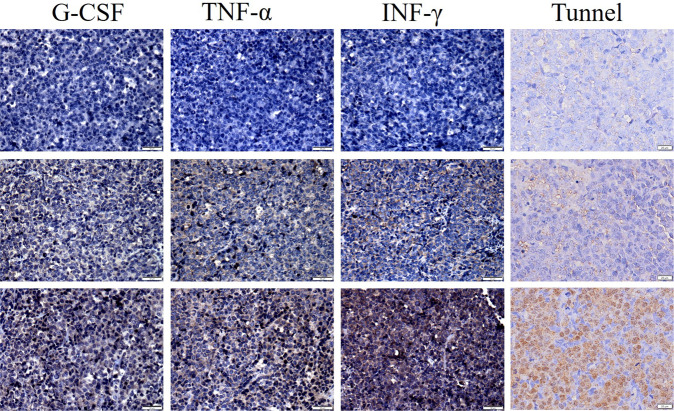
Immunohistochemical and tunnel assay of tumor tissue. Tumors were collected from Kappas-299 xenograft mice treated with saline (top), NC CAR T (middle) or CD30 CAR T (bottom). Formalin-fixed, paraffin-embedded tumor sections were consecutively cut and stained. (bar of G-CSF, TNF-α, INF-γ is 50 μm, a bar of the tunnel is 20 μm).

To test whether 9C11-2 CAR T cells exhibit antitumor efficacy toward CD30-positive human PTCL tumors in vivo, we established a Karpas 299 xenograft NCG mouse model. The results showed that CD30 CAR T cells efficiently suppressed tumor growth after day 9 and achieved a higher rate of tumor regression in the 9C11-2 CAR T group at day 30 (Fig. 5A–C). Furthermore, two xenograft NCG mice even have transient tumor regression periods in the 9C11-2 CAR T group (data not shown). On the other hand, NC CAR T cells suppressed tumor growth moderately and saline failed to suppress tumor growth. In contrast to this result, the mice treated with NC CAR T or saline showed a slight regression in tumor size, which led to the short-term survival of the mice with tumor progression (Fig. 5D). Furthermore, The mice treated with CD30 CAR T cells had significantly prolonged survival time compared with mice treated with NC CAR T cells or saline. Moreover, all mice injected with the CD30 CAR T cells survived more than 30 days; the median survival time was 55 days, while the median survival duration of the NC CAR T cells and saline-treated mice were 35 and 38 days, respectively (Fig. 5). There was no significant difference in weight change during the therapy (Fig. S4). The cytokine levels in the tumors were evaluated by IHC; the result showed that the CD30 CAR T (9C11-2) group had significantly higher levels of TNF-α, INF-γ, and GM-CSF than the NC CAR T or saline groups (Fig. 5E and Fig. 6). Furthermore, the apoptosis assay showed CD30 CAR T (9C11-2) group has more borrow stain than NC CAR T or saline groups (Fig. 6). These results may due to an increased tumor cell killing activity of CD30 CAR T cells compared to the NC CAR T cells and the saline group.

## Discussion

Peripheral T-cell lymphoma is a type of malignant tumor that originates from mature T cells. Currently, cyclophosphamide, doxorubicin, vincristine, and prednisone (CHOP regimen) are used to treat PTCLs. These treatment regimens are not satisfactory for most patients with PTCL since the clinical manifestations of PTCL are highly heterogeneous and aggressively malignant. At present, there is no optimal treatment strategy for PTCL, and chemotherapy based on CHOP (cyclophosphamide, doxorubicin, vincristine, and prednisone) for PTCL patients is often ineffective [29, 30]. The 5-year survival rate for patients with PTCL is less than 30%, which is significantly lower than other diffuse forms of lymphoma. The incidence of PTCL has been increasing year by year and based on the current treatment strategy, new and effective methods and strategies for treating PTCL are urgently needed.

In recent years, CAR T immunotherapy has demonstrated certain clinical benefits in hematologic tumor treatment. The tumor immunotherapy technology of the CAR-T cell therapy, which was first proposed in 1989 by the work of Gross et al., showed certain clinical benefits [31]. The recent study result shows that 108 patients treated with CD19 CAR T cells after chemotherapy, 42% of them continued to be in remission and the highest achieved ORR was 82% [32]. Moreover, CD19 CAR T-cell therapy has significantly improved the outcome for a 54-year-old man with relapsed/refractory large B-cell lymphoma, resulting in CR that is ongoing after 8 months of follow-up [33]. These results may due to the ability of CAR T cells to successfully target lymphoma cells. Similar to the CD19 CAR T therapy in leukemia and B cell lymphoma, CD30 is highly expressed in many kinds of PTCLs, and they are a potential target for PTCL CAR T therapy. Recently, a large number of studies have demonstrated the high expression of CD30 in PTCL. The results of some studies have shown that the high expression of CD30 was present in almost half of PTCL patients [24].

In this study, we recombinatorily expressed the CD30 protein fragment using tumor tissue from CD30-positive PTCL patients and prepared the monoclonal antibody against the CD30 protein fragment; the hope was that the CD 30 mAb would be compatible with the PTCL patients that were positive for CD30. The scFv segment of CD 30 mAb was amplified from the hybridoma cell line that was established from spleen cells of a recombinant CD30 protein immunized mouse. The IFA and IHC experiment demonstrates that this CD30 scFv could specifically target CD30-positive Karpas 299 cells and CD30-positive tumor-bearing tissue. This scFv segment was obtained from a CD30 positive patient and it more efficiently targeted CD30-positive tumors. Therefore, the new CD30 CAR (containing CD3ζ, 4-1BB, and CD30 scFv domains) was employed to generate CAR T cells and may exert more potent anti-cancer effects.

Immunotherapy has become one of the optional treatments for lymphoma in the clinic due to the excellent anti-tumor ability of this technology. The FDA has approved two anti-CD30 antibodies for the treatment of relapsed and refractory HL, and these drugs have obtained certain benefits in the clinic [34, 35]. There is sufficient evidence to show that CD30 may be an effective target for PTCL therapy. In this study, the CD30 specific CAR-T cell treatment was used to treat a CD30-positive tumor xenograft model. The result shows that CD30 CAR-T cells could effectively suppress tumor growth. However, the existence of mature T cells will still reduce the anti-cancer efficiency of CD30 CART cells. Moreover, CD30 is strongly expressed on peripheral T lymphoma cells, the CD30 CART cells tend to target them before targeting mature T cells with weak CD30 expression. Therefore, CAR-T cell therapy targeting CD30 for the treatment of refractory or recurrent CD30-positive PTCL could become a clinical application in the future.

## Conclusion

The CD30 CAR T cells exhibited an efficient cytotoxic effect after being cocultured with the target Karpas cells based on LDH, cell apoptosis, and RTCA assays. The CD30 CAR T cells also exhibited a significant tumor-inhibiting effect after being intravenously injected into PTCL xenograft tumors. These results suggest a new strategy in the field of PTCL therapy and we believe that anti CD30 CAR T may be used as primary therapy in certain circumstances.

## Supplementary information

Supplemental material

Dataset 1
