# Supplementary material for: A new immunotherapy strategy targeted CD30 in peripheral T-cell lymphomas: CAR-modified T-cell therapy based on CD30 mAb
Source: Cancer Gene Ther. 2021 Jan 29;29(2):167–77. doi: 10.1038/s41417-021-00295-8 (PMC8850188; doi:10.1038/s41417-021-00295-8)
Supplement: Supplementary file 1 — Supplemental material [file 41417_2021_295_MOESM1_ESM.docx]

**Supporting informations**

**A new immunotherapy strategy targeted CD30 in peripheral T cell lymphomas: CAR-modified T cell therapy based on CD30 mAb**

**Runing Title：A new CAR-T cell therapy based on CD30 mAb**

Yang Wu^1†^, Dan Chen^1†^, Ya Lu ^2^, Shu-chen Dong^3^, Rong Ma^1^, Wei-yan Tang^3^, Jian-qiu Wu^3^, Ji-feng Feng^3*^, Jian-Zhong Wu^1*^

^1^Research Center of Clinical Oncology, Jiangsu Cancer Hospital & Jiangsu Institute of Cancer Research & Nanjing Medical University Affiliated Cancer Hospital, Nanjing 210009, P. R. China

^2^Research Center of Clinical Oncology, Nanjing Medical University Affiliated Cancer Hospital, Nanjing 210009, P. R. China

^3^Department of Medical Oncology, Jiangsu Cancer Hospital & Jiangsu Institute of Cancer Research & Nanjing Medical University Affiliated Cancer Hospital, Nanjing 210009, P. R. China

Correspondence: Jian-Zhong Wu, wujzh@jszlyy.com.cn; Ji-feng Feng, feng_jifeng@sina.com

^†^These authors contributed equally to this work.

Table S1. The ELISA values of cultured supernatant of different hybridomas

| No. | 5E5 | 6B1 | 9C11 | 9F5 | 9G9 | 10D6 | 11C3 | 14C6 | 14D8 | 14D10 | 14E6 | 14E7 |
| --- | --- | --- | --- | --- | --- | --- | --- | --- | --- | --- | --- | --- |
| OD value | 0.727 | 0.808 | 2.495 | 0.813 | 0.761 | 1.651 | 0.722 | 1.275 | 1.293 | 1.601 | 1.324 | 1.374 |

Table S2. The sequencing results of three different 9C11 scFv.

|  | VH | Linker | VK |
| --- | --- | --- | --- |
| 9C11-1 | GAGGTGAAACTGCAGCAGTCTGGACCTGGCCTGGTGGCACCCTCACAGAGCCTGTCCATCACATGCACTGTCTCTGGGTTCTCATTATCCAGATATAGTGTACACTGGGTTCGCCAGCCTCCAGGAAAGGGTCTGGAGTGGCTGGGAATGATATGGGGTGGTGGAATCACAGACTATAATTCAGCTCTCAAATCCAGACTGAGCATCAACAAGGACAACTCCAAGAGCCAAGTTTTCTTAAAAATGAACAGTCTGCAAACTGATGACACAGCCATATACTACTGTGCCAGAAAGTATGGGTTGGATTACGACGGTGCTATGGACTACTGGGGCCAAGGGACCACGGTCACCGTCTCCTCA | GGTGGTGGTGGTTCTGGTGGTGGTGGTTCTGGCGGCGGCGGCTCC | GACATCCAGATGACCCAGTCTCCCAAATCCATGTCCATGTCAGTAGGAGAGAGGGTCACCTTGAGCTGCAAGGCCACTGACAATGTGCATACTTATGTATCCTGGTATCAACAAAAACCAGAGCAGTCTCCTAAACTGCTGATATACGGGGCATCCAACCGGTACACTGGGGTCCCCGATCGCTTCACAGGCAGTGGATCTGAAACAGATTTCACTCTGACCATCAGCAGTGTGCAGGCTGAAGACCTTGCAGATTATCACTGTGGACAGAGTTACAGGTATCCGCCCACGTTCGGTGCTGGGACCAAGCTGGAGCTGAAA |
| 9C11-2 | GAGGTGAAACTGCAGCAGTCTGGACCTGGCCTGGTGGCACCCTCACAGAGCCTGTCCATCACATGCACTGTCTCTGGGTTCTCATTATCCAGATATAGTGTACACTGGGTTCGCCAGCCTCCAGGAAAGGGTCTGGAGTGGCTGGGAATGATATGGGGTGGTGGAATCACAGACTATAATTCAGCTCTCAAATCCAGACTGAGCATCAACAAGGACAACTCCAAGAGCCAAGTTTTCTTAAAAATGAACAGTCTGCAAACTGATGACACAGCCATATACTACTGTGCCAGAAAGTATGGGTTGGATTACGACGGTGCTATGGACTACTGGGGCCAAGGGACCACGGTCACCGTCTCCTCA | GGTGGTGGTGGTTCTGGTGGTGGTGGTTCTGGCGGCGGCGGCTCC | GACATCCAGATGACCCAGTCTCCCAAATCCATGTCCATGTCAGTAGGAGAGAGGGTCACCTTGAGCTGCAAGGCCACTGACAATGTGCATACTTATGTATCCTGGTATCAACAAAAACCAGAGCAGTCTCCTAAACTGCTGATATACGGGGCATCCAACCGGTACACTGGGGTCCCCGATCGCTTCACAGGCAGTGGATCTGAAACAGATTTCACTCTGACCATCAGCAGTGTGCAGGCTGAAGACCTTGCAGATTATCACTGTGGACAGAGTTACAGGTATCCGCTCACGTTCGGTGCTGGGACCAAGCTGGAGCTGAAA |
| 9C11-3 | GAGGTGAAACTGCAGCAGTCTGGACCTGGCCTGGTGGCACCCTCACAGAGCCTGTCCATCACATGCACTGTCTCTGGGTTCTCATTATCCAGATATAGTGTACACTGGGTTCGCCAGCCTCCAGGAAAGGGTCTGGAGTGGCTGGGAATGATATGGGGTGGTGGAATCACAGACTATAATTCAGCTCTCAAATCCAGACTGAGCATCAACAAGGACAACTCCAAGAGCCAAGTTTTCTTAAAAATGAACAGTCTGCAAACTGATGACACAGCCATATACTACTGTGCCAGAAAGTATGGGTTGGATTACGACGGTGCTATGGACTACTGGGGCCAAGGGACCACGGTCACCGTCTCCTCA | GGTGGTGGTGGTTCTGGTGGTGGTGGTTCTGGCGGCGGCGGCTCC | GAAATTGTGTTGACGCAGTCTCCTGCTTCCTTAGCTGTATCTCTGGGGCAGAGGGCCACCATCTCATACAGGGCCAGCAAAAGTGTCAGTACATCTGGCTATAGTTATATGCACTGGAACCAACAGAAACCAGGACAGCCACCCAGACTCCTCATCTATCTTGTATCCAACCTAGAATCTGGGGTCCCTGCCAGGTTCAGTGGCAGTGGGTCTGGGACAGACTTCACCCTCAACATCCATCCTGTGGAGGAGGAGGATGCTGCAACCTATTACTGTCAGCACATTAGGGAGCTTACACGTTCGGAGGGGGGACCAAGCTGGAAATCAAAC |

Table S3. The amino acid sequences of complementary regions of 9C11 scFv.

|  | CDR1 | CDR2 | CDR3 |
| --- | --- | --- | --- |
| 9C11VH | GFSLSRYS | IWGGGIT | ARKYGLDYDGAMDY |
| 9C11VK-1 | DNVHTY | GAS | GQSYRYPPT |
| 9C11VK-2 | DNVHTY | GAS | GQSYRYPLT |
| 9C11VK-3 | KSVSTSGYSY | LVS | QHIRELTR |


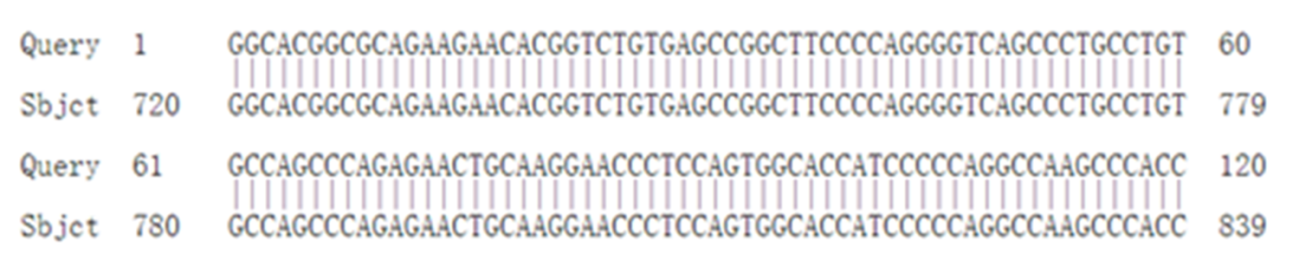


Figure S1. The sequencing result of the pET328a-CD30 plasmid.


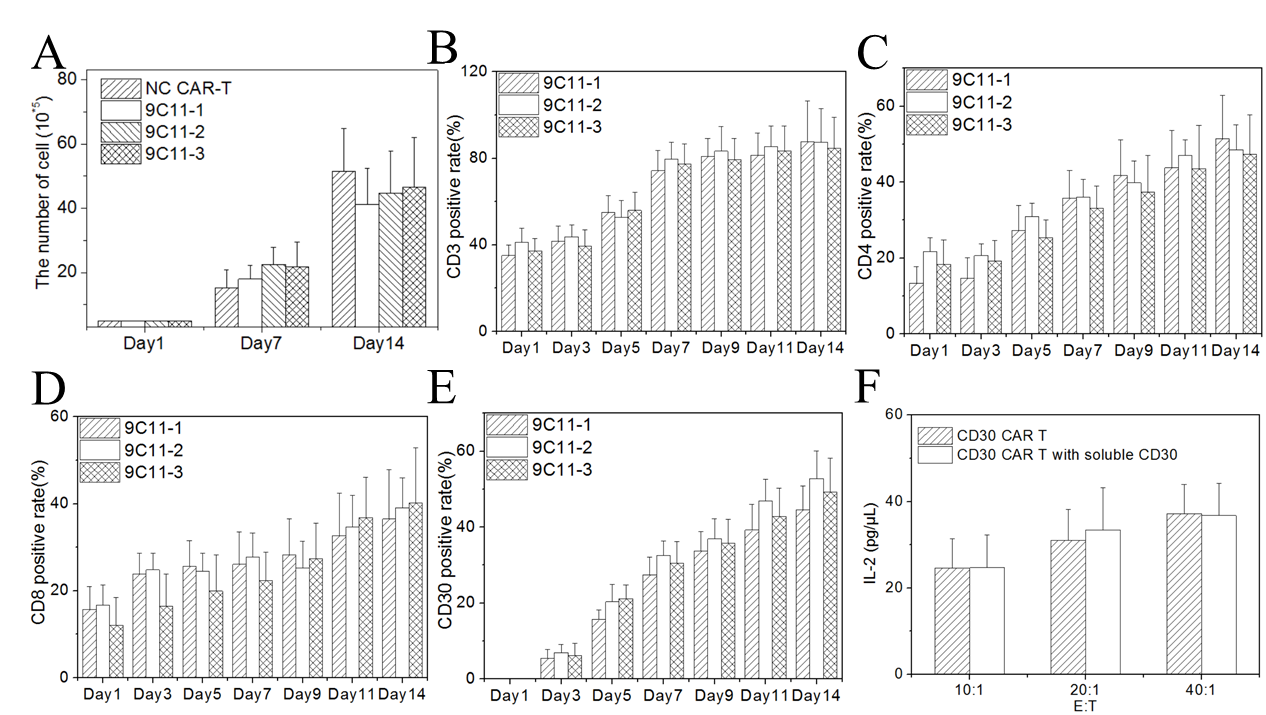


Figure S2. The generation and proliferation of three kinds of CD30 CAR T cells (A-E), soluble CD30 antigen block experiment (F), each example was tested three times.


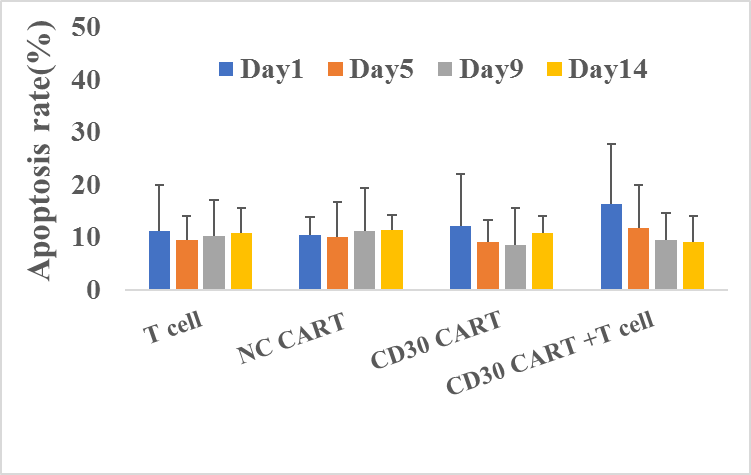


Figure S3. The apoptosis rate of T cell, NC CART, CD30 CART and CD30 CART+ T cell during 14 culture days, each example was tested three times.


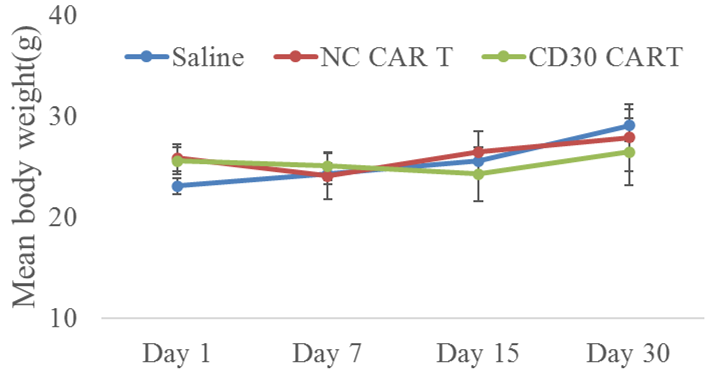


Figure S4. The body weight change of Kappas-299 xenografts mice treated with saline, NC CAR T or CD30 CAR T.
