# Supplementary figures and images for: A new immunotherapy strategy targeted CD30 in peripheral T-cell lymphomas: CAR-modified T-cell therapy based on CD30 mAb
Source: Cancer Gene Ther. 2021 Jan 29;29(2):167–77. doi: 10.1038/s41417-021-00295-8 (PMC8850188; doi:10.1038/s41417-021-00295-8)

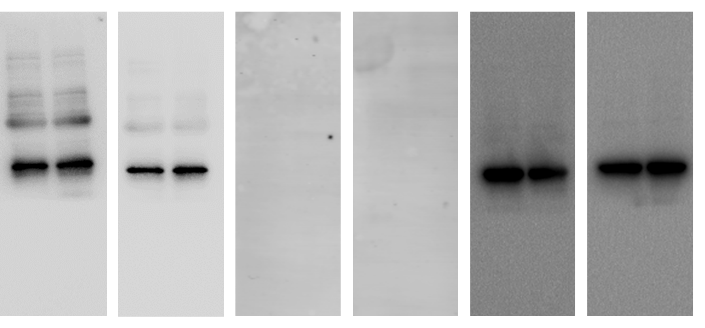

Supplement: Supplementary file 2 — Dataset 1 [file 41417_2021_295_MOESM2_ESM.docx]
